# Supplementary material for: Enzymatic measurement of short-chain fatty acids and application in periodontal disease diagnosis
Source: PLoS One. 2022 Jul 15;17(7):e0268671. doi: 10.1371/journal.pone.0268671 (PMC9286277; doi:10.1371/journal.pone.0268671)
Supplement: S1 Table — (DOCX) [file pone.0268671.s001.docx]

**S1 Table.** Biochemical properties of recombinant His-tagged butyrate kinase from *Thermosediminibacter oceani* (DSM 16646)

| Parameter | Results |
| --- | --- |
| Molecular mass |  |
| SDS-PAGE (Da) | 39,000 |
| Calculated (Da) | 39,088 |
| Calculated isoelectric point (pI) | 4.99 |
| pH stability ^a^ | 5–11 |
| pH optimum ^b^ | 7–8.5 |
| Thermostability (˚C) ^c^ | 70 |
| Specific activity (U/mg) ^d^ | 513 |

a: Aliquots of 1 U enzyme per mL in the following buffer systems (all at 100 mM) containing 0.1% TN-100 were incubated for 5 h at 37 °C; acetate-NaOH (pH 5–6), MES-NaOH (pH 6–7), PIPES-NaOH (pH 7–7.5), Tris-HCl (pH 7–9), CHES-NaOH (pH 9–10), and CAPS-NaOH (pH 10–11). After incubation, the remaining activity was analyzed.

b: The following buffer systems were used to determine the optimum pH: acetate-NaOH (pH 5–6), MES-NaOH (pH 6–7), PIPES-NaOH (pH 7–7.5), Tris-HCl (pH 7–9), CHES-NaOH (pH 9–10), and CAPS-NaOH (pH 10–11).

c: Aliquots of 1 mg enzyme per mL in 50 mM potassium phosphate (pH 7) containing 0.1% TN-100 were incubated in sealed tubes for 30 min at 4–80˚C. After incubation, all tubes were rapidly cooled in an ice bath and analyzed for activity.

d: Protein levels were determined using the Bradford dye-binding method using the Bio-Rad protein assay kit (Hercules, CA, USA). Bovine serum albumin was used as the assay protein standard.

SDS-PAGE, sodium dodecyl sulfate-polyacrylamide gel electrophoresis
