# Supplementary figures and images for: Enzymatic measurement of short-chain fatty acids and application in periodontal disease diagnosis
Source: PLoS One. 2022 Jul 15;17(7):e0268671. doi: 10.1371/journal.pone.0268671 (PMC9286277; doi:10.1371/journal.pone.0268671)

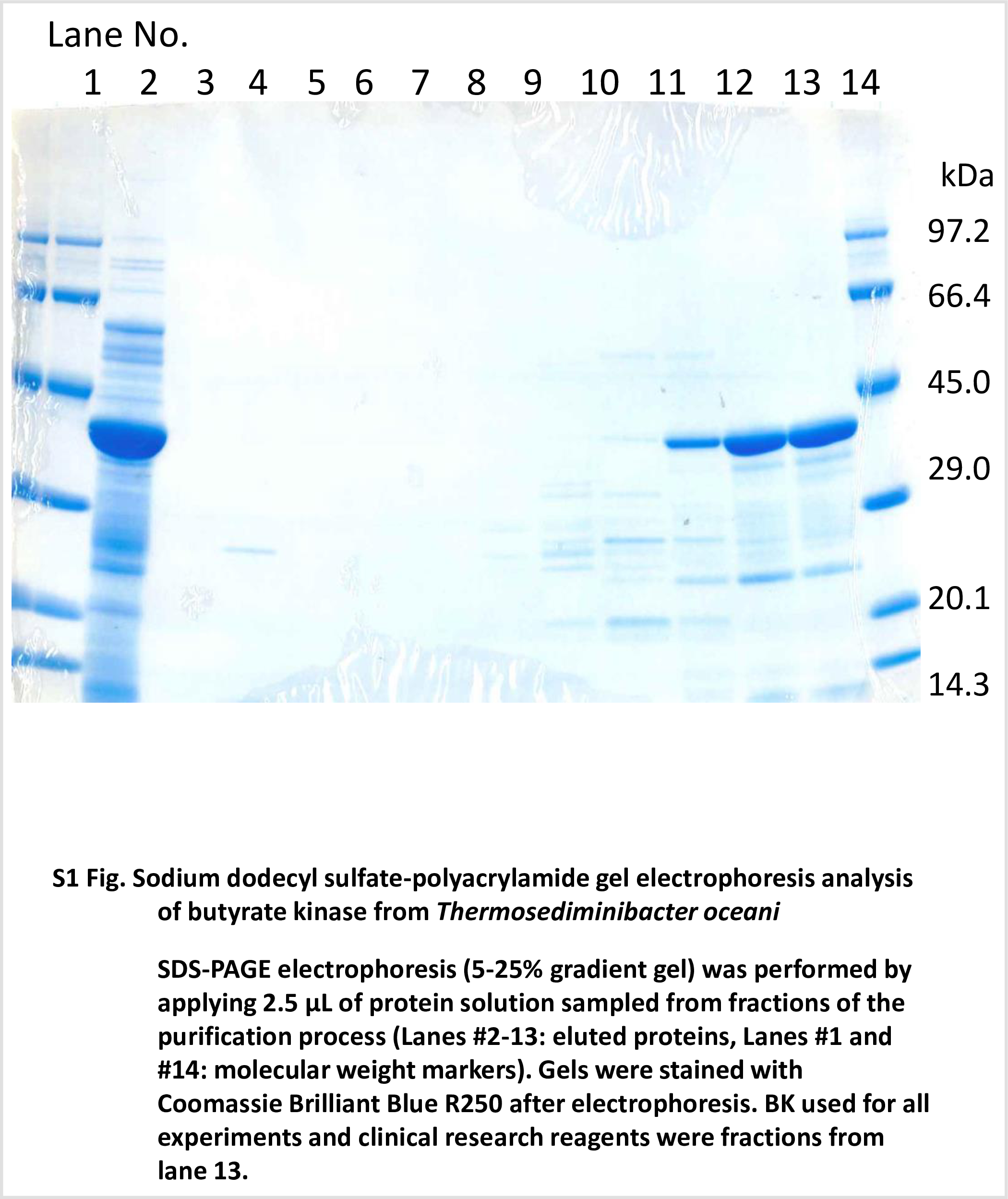

Supplement: S1 Fig — (TIF) [file pone.0268671.s002.tif]

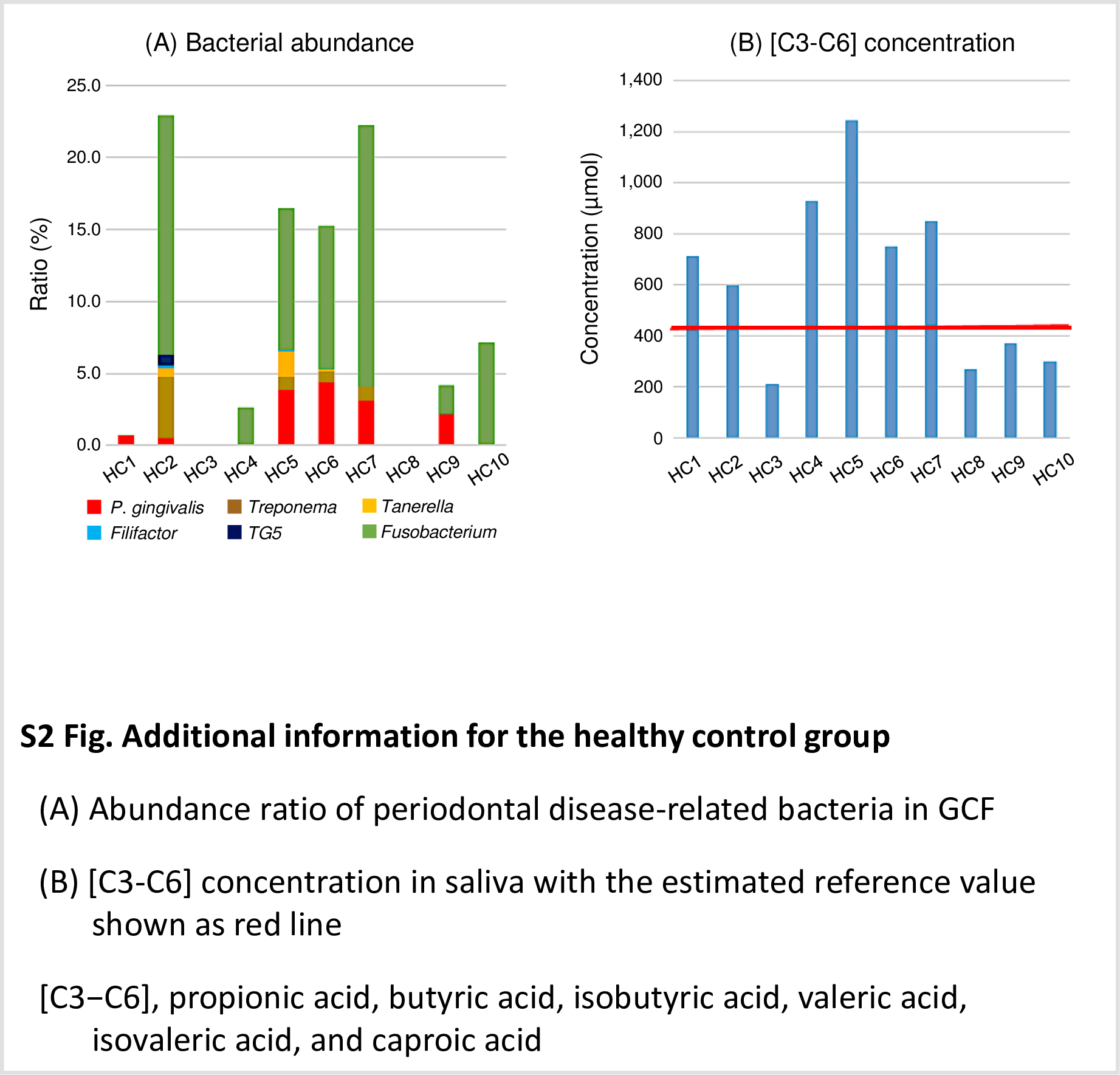

Supplement: S2 Fig — (TIF) [file pone.0268671.s003.tif]

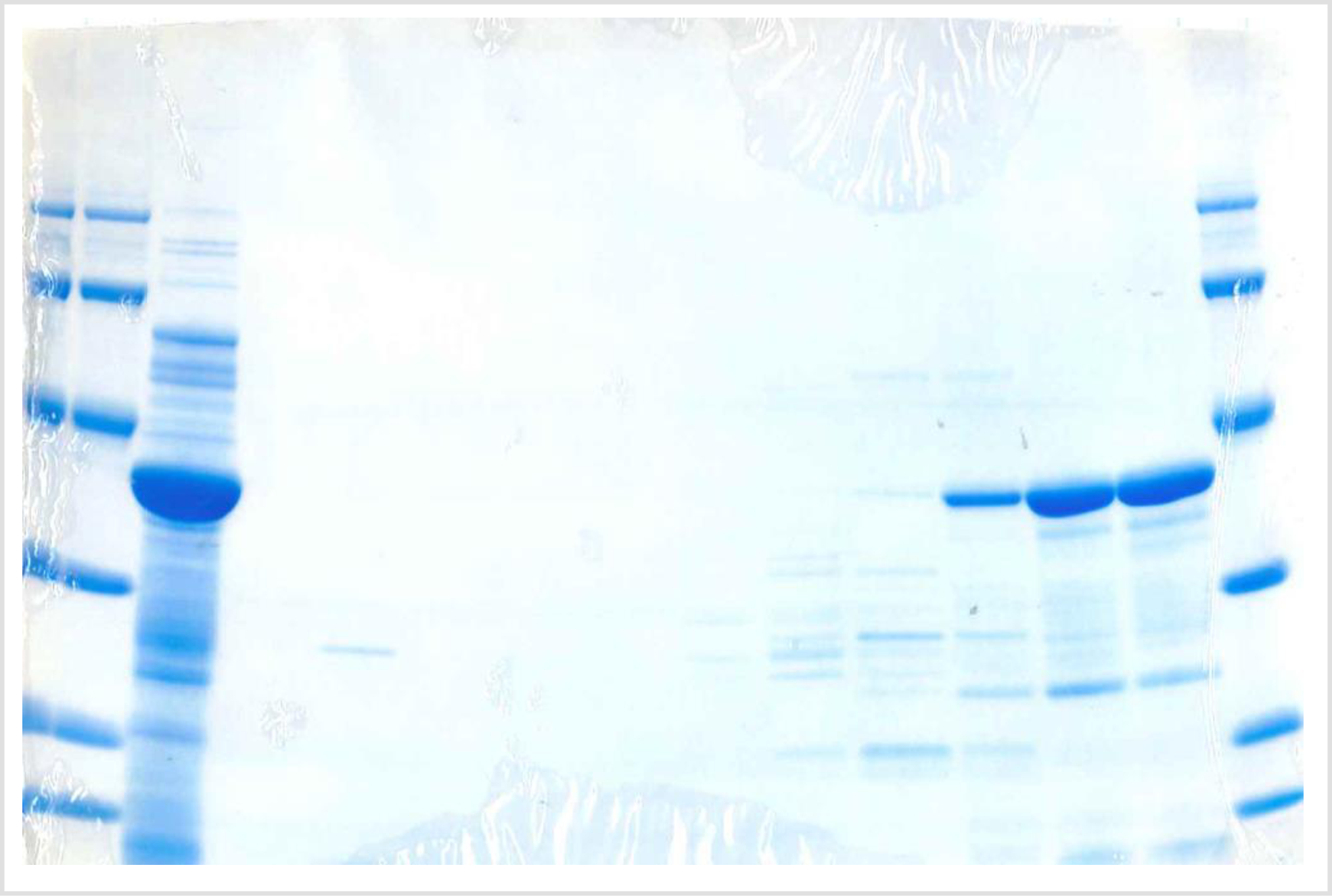

Supplement: S1 Raw image — (TIF) [file pone.0268671.s004.tif]
